# Relationship between the efficacy of immunotherapy and characteristics of specific tumor mutation genes in non‐small cell lung cancer patients

**DOI:** 10.1111/1759-7714.13447

**Published:** 2020-04-27

**Authors:** Peng Song, Dongliang Yang, Hanping Wang, Xiaoxia Cui, Xiaoyan Si, Xiaotong Zhang, Li Zhang

**Affiliations:** ^1^ Department of Respiratory Medicine Peking Union Medical College Hospital, Chinese Academy of Medical Science & Peking Union Medical College Beijing China; ^2^ Department of General Education Courses Cangzhou Medical College Beijing China

**Keywords:** Immune checkpoint inhibitor, *KRAS*, non‐small cell lung cancer, PD‐1, PD‐L1

## Abstract

**Background:**

Immune checkpoint inhibitors (ICIs) have greatly improved the prognosis and overall management of non‐small cell lung cancer (NSCLC) patients, but in the long term less than 20% of patients benefit from treatment with ICIs. Therefore, it is necessary to guide the choice of immunotherapy population through biomarkers in order to maximize the benefit for NSCLC patients. This article mainly explores the relationship between the efficacy of immunotherapy and specific tumor mutation gene characteristics in an NSCLC population.

**Methods:**

This was a prospective analysis of patients with advanced NSCLC who visited the Department of Respiratory Medicine of Peking Union Medical College Hospital from March 2018 to June 2019 and were instructed to use PD‐1 inhibitors. The follow‐up deadline was 31 December 2019. The tumor pathological tissues were tested for tumor mutation genes, and the patients were evaluated for efficacy according to RECIST 1.1. The patients were divided into the durable benefit group (DCB) and the nonsustainable benefit group (NDB). DCB/NDB was used as the outcome variable. Various statistics methods were used to explore the independent predictors of long‐term benefits associated with immunotherapy and to draw a progression‐free survival curve for the relevant predictors.

**Results:**

A total of 44 patients were examined for tumor mutation genes in pathological tissues; 20 in the DCB group and 24 in the NDB group. Specific gene mutations occurred in *TP53* 38.64%, *KRAS* 31.82%, *EGFR* 20.45%, *BRCA* 20.45%, *ERBB* (excluding *EGFR*) 18.18%, *PTEN* 15.91%, *CDK4/6* 13.64%, *POLE* 11.36%, *MET* 11.36%, *PIK3CA* 9.10%, *FGFR* 9.10%, *BRAF* 9.10%, *JAK* 9.10%, *ALK* 6.82%, *POLD1* 4.55%, *BLM* 4.55%. Chi‐square test results showed that there were statistically significant differences between DCB and NDB groups with eight mutations such as *KRAS*. Logistic regression showed that the *KRAS* mutation was statistically significant (*P* < 0.001). Two accuracy indicators, Random Forest Classification of Mean Decrease Gini and Mean Decrease Accuracy, evaluated the importance of the impact of different gene mutations on the outcome. Under two different measures, the variables were all *KRAS* mutations. It is suggested that the mutation of the *KRAS* gene is an independent predictor of the long‐term benefit of immunotherapy.

**Conclusions:**

The mutation of *KRAS* gene in tumor tissues is an independent predictor of the long‐term benefit of immunotherapy, and the predictive ability is better.

## Introduction

Non‐small cell lung cancer (NSCLC) accounts for 85% of diagnosed lung cancers. About 50% of NSCLC patients are diagnosed when they are already in stage IV, and their five‐year survival rate is less than 10%.^1^ The emergence of immune checkpoint inhibitors (ICIs) targeting programmed cell death 1 (PD‐1) or its ligand (PD‐L1) has significantly changed the treatment and management of locally advanced and advanced NSCLC. Multiple randomized controlled trials (RCTs) have shown that ICIs are superior to docetaxel^2^, ^3^, ^4^ as a second‐line treatment for advanced NSCLC patients. ICIs have been approved by the US Food and Drug Administration (FDA) to treat patients with 15 different cancer types.^5^ However, most tumors appear to lack T cell infiltration and active expression of immune genes. Only 20% of patients with advanced NSCLC benefit from the treatment, whereas up to 50% of patients experience treatment‐related adverse events (AEs). Considering the high cost of the drug, the limited population that benefits from it, and the potential for serious side effects, it is important to explore biomarkers for selecting patients with advanced NSCLC who might benefit from ICI treatment.

Recent studies have found that genetic changes in specific driver genes activate tumor cell proliferation, thereby supporting tumor growth. It has been shown that certain oncogenic pathways also affect the immune system's recognition of tumors, especially T cell‐mediated recognition. Smoking‐related *KRAS* mutations are the most common carcinogenic change in NSCLC.^6^, ^7^ Recent clinical evidence indicates that tumors classified as KRAS‐TP53 have an immunogenic phenotype and may be more sensitive to nivolumab.^8^

This study examined tumor mutation genes in the pathological tissues of 44 Chinese NSCLC patients treated with anti‐programmed death (PD)‐1 monoclonal antibodies (including pembrolizumab, nivolumab, and sintilimab) to identify genetic changes associated with the clinical benefit of immune checkpoint inhibitors (ICIs). The goal of the study is to accurately select the population that will benefit from immunotherapy.

## Methods

### Patients

A prospective analysis was conducted of patients with advanced NSCLC who visited the Peking Union Medical College Hospital from March 2018 to June 2019 and were instructed to use PD‐1/PD‐L1 inhibitors. According to the solid tumor response evaluation standard (Response Evaluation Criteria in Solid Tumors (RECIST) version 1.1), there are four categories consisting of complete response (CR), partial response (PR), stable disease (SD), and progressive disease (PD). Durable clinical benefit (DCB) is defined as CR, PR, or SD lasting more than six months. Patients who developed disease progression within six months were classified as having no durable benefit (NDB). Efficacy is determined every six to eight weeks after the start of the immunotherapy. In special cases, the time interval can be adjusted to suit the patients' needs. The enrollment deadline for patients was 30 June 2019, and the follow‐up deadline was 31 December 2019. The Ethics Committee of the Peking Union Medical College Hospital has approved this study, which is in line with the ethical principles of the Helsinki Declaration. All patients have signed informed consent.

### Sample collection

Fresh tissue was sampled to detect gene mutation before immunotherapy, or a pathological white section of tumor tissue was used that was obtained within two years before treatment with PD‐1/PD‐L1 inhibitor. It is necessary to note the time of tumor tissue ex vivo; section requirements: tumor cells > 20%, area > 10 × 10 mm, thickness of 5–10 μm, and 15 slices or more.

### Main experimental reagents and instruments

Tissue genomic DNA extraction kit DP304 (TIANGEN), KAPA HyperPlus Kits (Roche), HyperCap Bead Kit (Roche), SureSelect Target Enrichment Kit ILM Indexing Hyb Module Box 2 (Agilent), PlateLoc Thermal Microplate Sealer (Agilent), Herculase II Fusion DNA Polymerase Kit (Agilent), Sequencing and Library Building Platform (IIIumina USA) were used.

### Experimental method

(i) Fresh tumor tissue was processed with quality control; (ii) DNA extraction of formalin‐fixed paraffin‐embedded (FFPE) samples was performed using the GeneRead DNA FFPE Tissue Kit; (iii) plasma and leukocytes were separated from peripheral blood samples; (iv) extraction of free DNA from peripheral blood: HiPure Circulating DNA kits were used to extract free DNA; (v) blood/cell/tissue genomic DNA extraction kit (DP304) was used to extract leukocyte DNA (germline DNA); (vi) a DNA library was established using KAPA Biosystems HyperPlus Kits to build the library; (vii) probe hybridization was performed for 642 gene panels（Appendix S1） with the Hyper Cap Target Enrichment Kit and SeqCap EZ Probes; (viii) full exon probe hybridization was performed using Agilent probes and related kits; (ix) to mix and dilute different libraries so that the DNA concentration in all libraries was 10 nM, and the total volume of the system is 20 μL; (x) online sequencing was performed using the Illumina HiSeq X Ten high‐throughput sequencing platform.

### Statistical analysis

Descriptive analysis was used to summarize genetic characteristics. Fisher's exact test was used to study the association between mutations and DCB/NDB. DCB/NDB was used as the outcome variable, and specific gene mutations were used as independent variables for lasso regression, logistic regression, and machine learning random forest analysis to explore independent predictors related to the long‐term benefits of immunotherapy. The Kaplan‐Meier method was used to draw the progression‐free survival curve of NSCLC immunotherapy patients with or without specific gene mutations. A receiver operating characteristic (ROC) curve was drawn to evaluate the prediction ability.

## Results

### Tumor mutant gene characteristics

This prospective study included 63 patients with advanced NSCLC who received PD‐1 monoclonal antibody therapy, and 44 patients who could eventually provide sufficient tissue samples for the gene panel testing. Of these 44 patients, 66.15% were given pembrolizumab, 26.15% sindilizumab, and 7.69% nivolumab. The objective response rate (ORR) with PD‐1 was 43.18%, and the disease control rate (DCR) was 72.73%. The DCB group accounted for 43.18% of patients, and the NDB group accounted for 56.82%. The top 16 gene mutations were selected for statistical analysis. The mutation rates were *TP53* 38.64%, *KRAS* 31.82%, *EGFR* 20.45%, *BRCA* 20.45%, *ERBB* (excluding *EGFR*) 18.18%, *PTEN* 15.91%, *CDK4/6* 13.64%, *POLE* 11.36%, *MET* 11.36%, *PIK3CA* 9.10%, *FGFR* 9.10%, *BRAF* 9.10%, *JAK* 9.10%, *ALK* 6.82%, *POLD1* 4.55%, and *BLM* 4.55%.

### Relationship between specific tumor gene mutations and possibility of DCB

Table 1 shows the correlation between DCB/NDB possibilities and molecular characteristics. Chi‐square test results showed a significant difference in the frequency of *KRAS* mutations (*P* < 0.001), *KRAS* + *TP53* combination mutation (*P* = 0.005), *TP53* + *PTEN* combination mutation (*P* = 0.036), *PTEN* mutation (*P* = 0.035), *JAK1/2/3* mutation (*P* = 0.030), *TP53* mutation (*P* = 0.042), *EML4‐ALK* mutation (*P* = 0.049), *pan‐ErbB* mutation (*P* = 0.039) between the DCB and NDB groups. The DCB group had higher rates of *KRAS* mutations, *KRAS* + *TP53* combination mutations, *TP53* + *PTEN* combination mutations, *PTEN* mutations, *JAK1/2/3* mutations, *TP53* mutations, and *EML4‐ALK* mutations, suggesting that patients with these genetic mutations may benefit from immunotherapy. The incidence of *ERBB* comutation in NDB was significantly higher than that in the DCB group, suggesting that the *ERBB* comutation may be a type that does not benefit immunotherapy. It is worth noting that the *EML4*‐*ALK* mutation, *JAK1/2/3* mutation, *KRAS* + *TP53* combination mutation, and the *TP53* + *PTEN* combination mutation only occurred in DCB patients, suggesting that these four gene mutations may be potential genes for effective immunotherapy. *BRCA* mutations, *CDK4/6* mutations, and *BLM* mutations had a higher incidence in the NDB group, but no statistical difference was shown due to the sample size, which indicates that if these gene mutations were used for immunotherapy, they would have poor curative effect, which requires further exploration.

**Table 1:** Association between the possibility of DCB/NDB and tumor‐specific gene mutations

Gene	NDB (*n* = 24)	DCB (*n* = 20)	*P*‐value
*Pan‐ErbB*			
1	12	4	**0.039**
0	12	16	
*KRAS*			
1	1	13	**<0.001**
0	23	7	
*BRCA*			
1	7	2	0.117
0	17	18	
*EML4‐ALK*			
1	0	3	**0.049**
0	24	17	
*PIk3CA*			
1	2	2	1.000
0	22	18	
*MET*			
1	3	2	1.000
0	21	18	
*TP53*			
1	6	11	**0.042**
0	18	9	
*FGFR*			
1	2	2	1.000
0	22	18	
*BRAF*			
1	2	2	1.000
0	22	18	
*CDK4/6*			
1	5	1	0.279
0	19	19	
*POLE*			
1	3	2	1.000
0	21	18	
*POLD1*			
1	1	1	1.000
0	23	19	
*BLM*			
1	2	0	0.493
0	22	20	
*JAK1/2/3*			
1	0	4	**0.030**
0	24	16	
*PTEN*			
1	1	6	**0.035**
0	23	14	
*TP53 + PTEN*			
1	0	4	**0.036**
0	24	16	
*KRAS + TP53*			
1	0	6	**0.005**
0	24	14	

Bold font means *P* value <0.05, suggesting statistical significance.

### Exploring independent predictors of outcomes for immunotherapy

**Figure 1:**
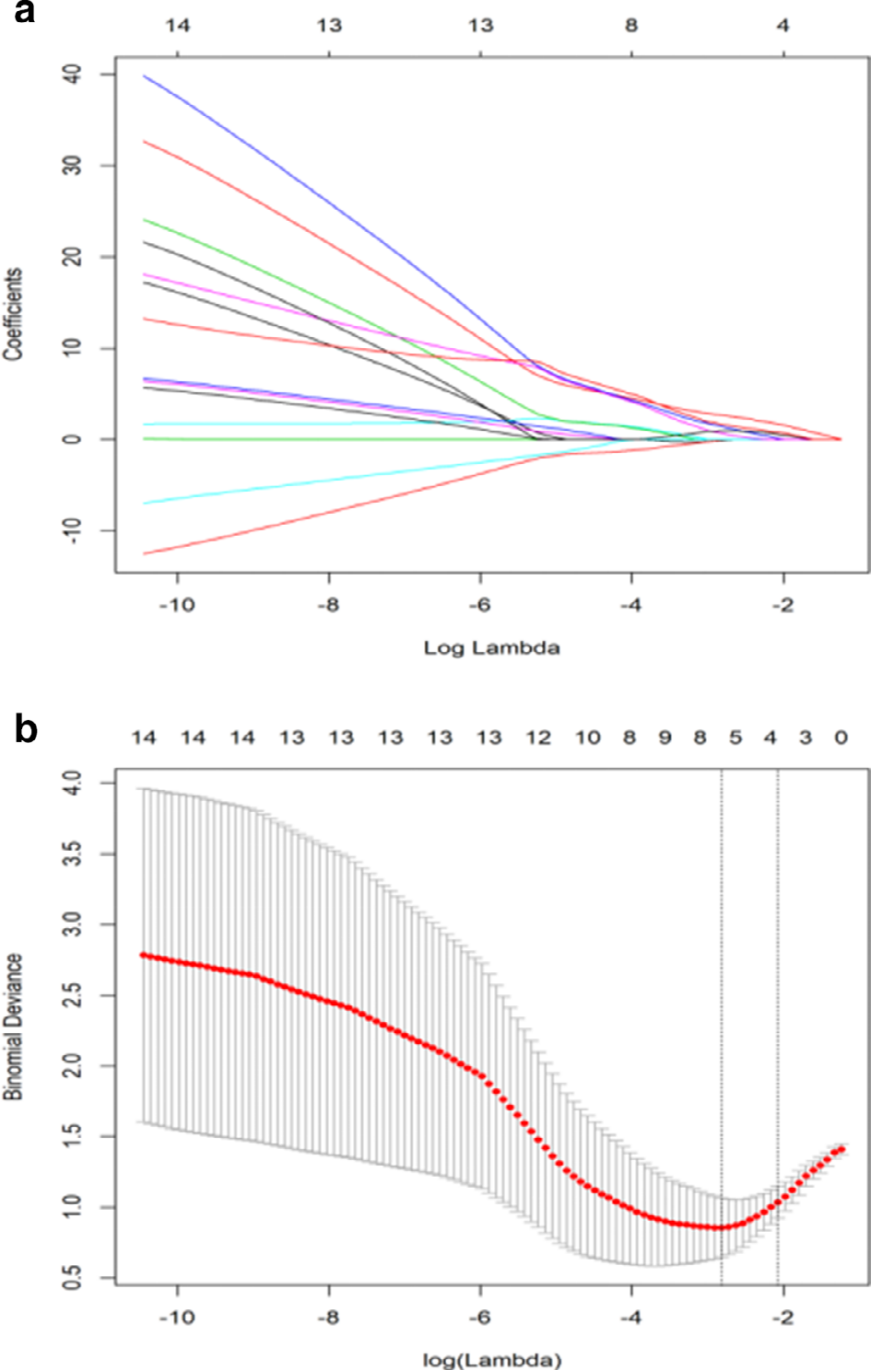
(**a**) Lasso coefficient curve for 17 independent variables; (**b**) log (Lambda) sequence coefficient distribution.

Lasso regression was performed with the long‐term benefit as the outcome variable and the genetic mutation as the independent variable. The glmnet package in R software was used. When the penalty parameter lambda = lambda.1se = 0.125 773 1 was used, the error of the model was relatively small. At this time, the nonzero gene mutations (Table 2, Fig 1a–b) retained in the model were *KRAS*, *EML4‐ALK*, *PTEN*, and *TP53* + *PTEN*. Among these, the ranking of *KRAS* > *TP53* + *PTEN* > *PTEN* > *EML4*‐*ALK* was conducted according to importance.

**Table 2:** Seventeen independent variable coefficients

Gene	Coefficient
(Intercept)	−1.0209753
*Pan‐ErbB*	
*KRAS*	1.696 336 5
*BRCA*	
*EML4‐ALK*	0.126 027 6
*PIK3CA*	
*MET*	
*TP53*	
*FGFR*	
*BRAF*	
*CDK4/6*	
*POLE*	
*POLD1*	
*BLM*	
*JAK1/2/3*	
*PTEN*	0.588 657 5
*TP53* + *PTEN*	0.816 064 3
*KRAS* + *TP53*	

In the multivariate analysis, *KRAS* mutations and *TP53* + *PTEN* mutations were included in logistic regression (due to sample size limitations), and the results showed that *KRAS* mutations were statistically significant (Table 3, *P* < 0.001). It is suggested that the mutation of the *KRAS* gene is an independent predictor of the long‐term benefit of immunotherapy. There was no significant statistical correlation between the *TP53* + *PTEN* combination mutation and long‐term benefit outcomes.

**Table 3:** Logistic regression analysis results

Estimate	Std.	Error	*P‐*value
(Intercept)	−2.0369	0.6138	<0.001
*KRAS*	3.8286	0.9799	<0.001
*TP53* + *PTEN*	20.6030	3261.3194	0.994 960

Unreliable conclusions are often obtained from logistic regression. Therefore, although logistic regression indicated that the *KRAS* gene mutation is an independent predictor of whether long‐term benefits are obtained, in order to form a closed loop of evidence, the random forest algorithm of machine learning was used to find important genes to compare with the results of the logistic regression analysis.

**Figure 2:**
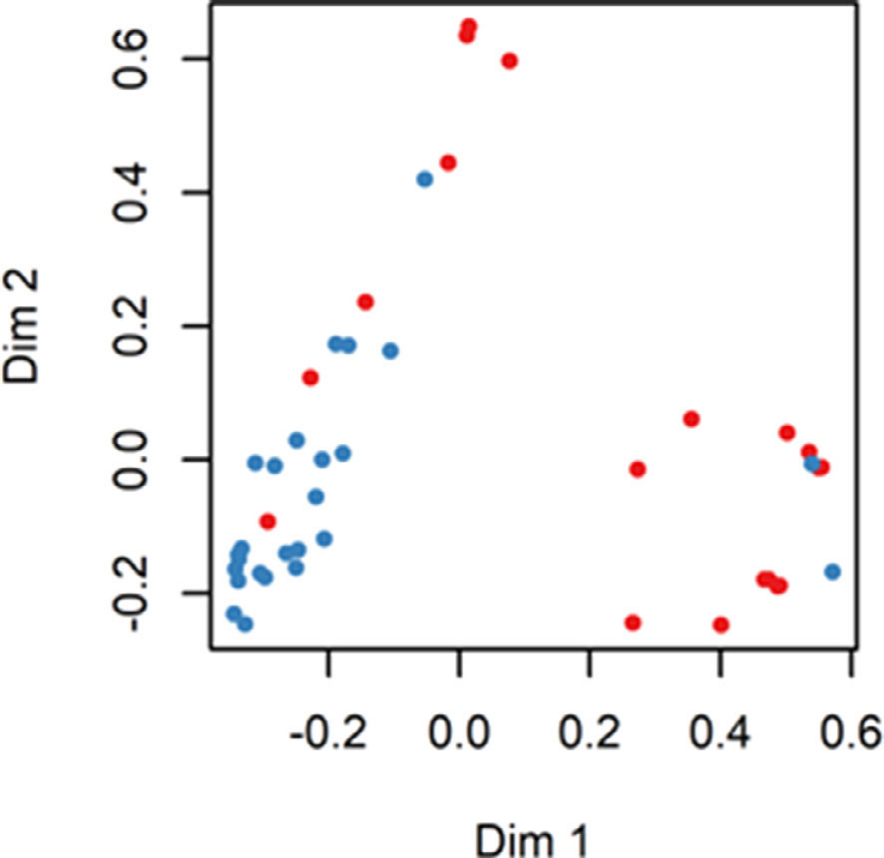
Multidimensional scale.

**Figure 3:**
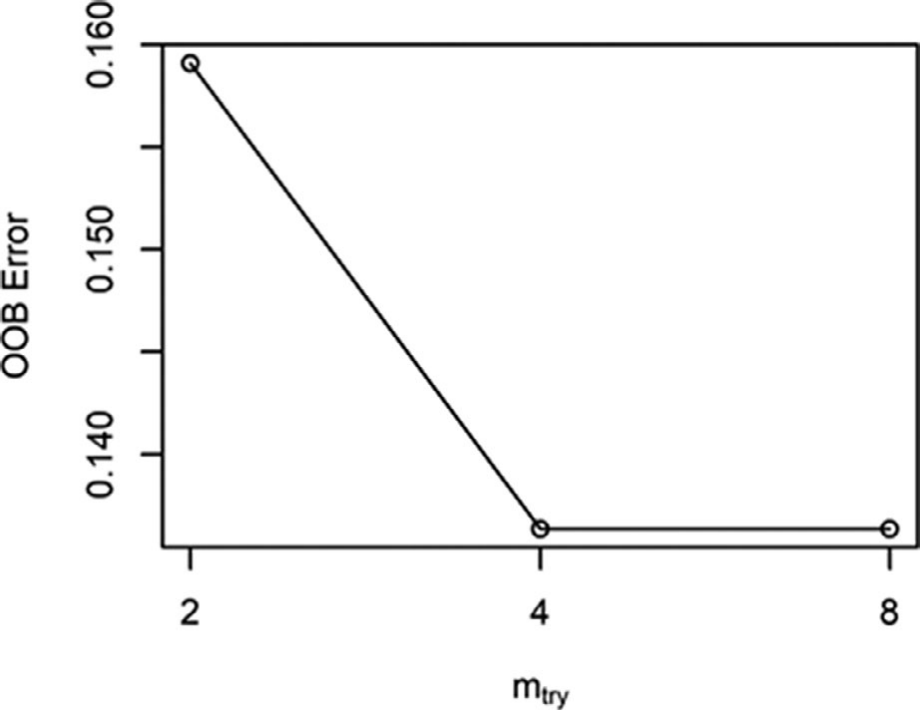
The relationship between parameters and errors.

**Figure 4:**
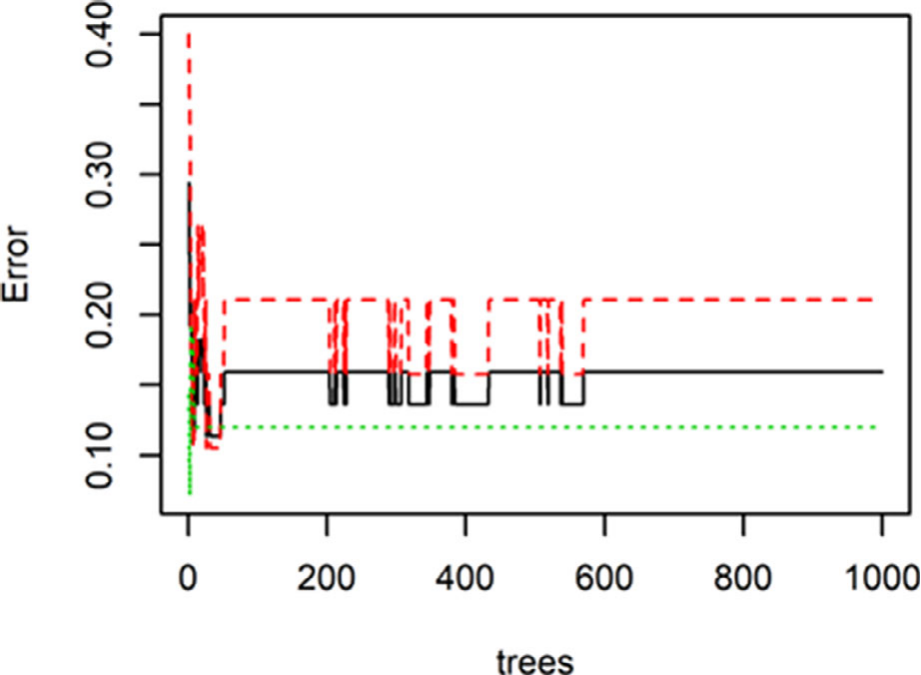
The relationship between the number of forests and errors.

Predictive models were built based on whether the patient's long‐term benefit is the dependent variable and genetic mutation is the independent variable. The R window was used to draw a multidimensional scale (MDS), as shown in Fig 2, and 24 NDB samples (blue dots) and 20 DCB samples (red dots) were divided into two distinct categories. The RandomForest package in R software was used to perform random forest classification on the data. To prevent overfitting, a portion of the data was selected as the training set and another portion of the sample was selected as the test set. The purpose of this was to obtain the relationship between parameters and errors (Fig 3) and the relationship between the number of forests and errors (Fig 4).

It can be seen from the two figures that when the number of trees reaches more than 600, the error rate gradually stabilizes. The most optimal parameters are mtry = 4 and ntree = 1000.The out‐of‐bag (OOB) estimate of the error rate is 13.64%, which is equivalent to a cross‐validation classification accuracy rate of 86.36%, and the original sample back‐generation prediction accuracy rate is 100%. This indicates that there is a stable correlation between the predictors of this study and the long‐term benefits of immune checkpoint inhibitor treatment.

**Figure 5:**
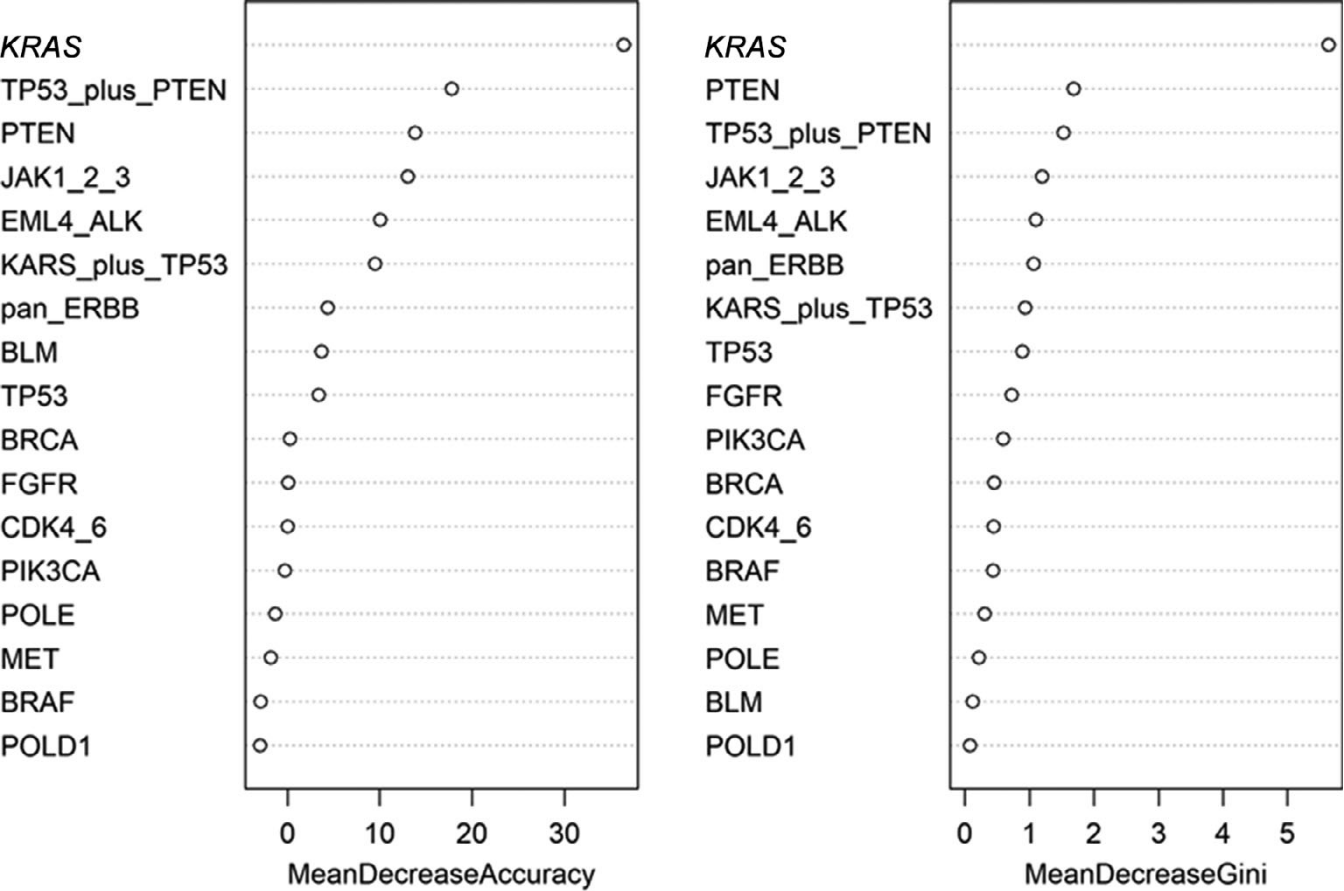
The ranking chart of the two accuracy indicators.

When the above parameters (mtry = 4, ntree = 1000) were fixed, the average minimum Gini index reduction (Mean Decrease Gini) and average accuracy decrease (Mean Decrease Accuracy) were calculated for each gene mutation. The purpose of this was to obtain a ranking chart (Fig 5) of the two accuracy indicators to evaluate the importance of the impact of different gene mutations on the outcome. The most important independent variable is the *KRAS* gene mutation, which ranks first. This shows that the *KRAS* gene is the most important gene in the classification algorithm.

*KRAS* gene mutations are the most important variables in lasso regression, logistic regression, and machine learning random forest algorithms. Evidence of closed loops has accumulated, suggesting that *KRAS* gene mutations are independent predictors of whether immunotherapy can durably benefit patients.

### Progression‐free survival curves with or without KRAS mutation

**Figure 6:**
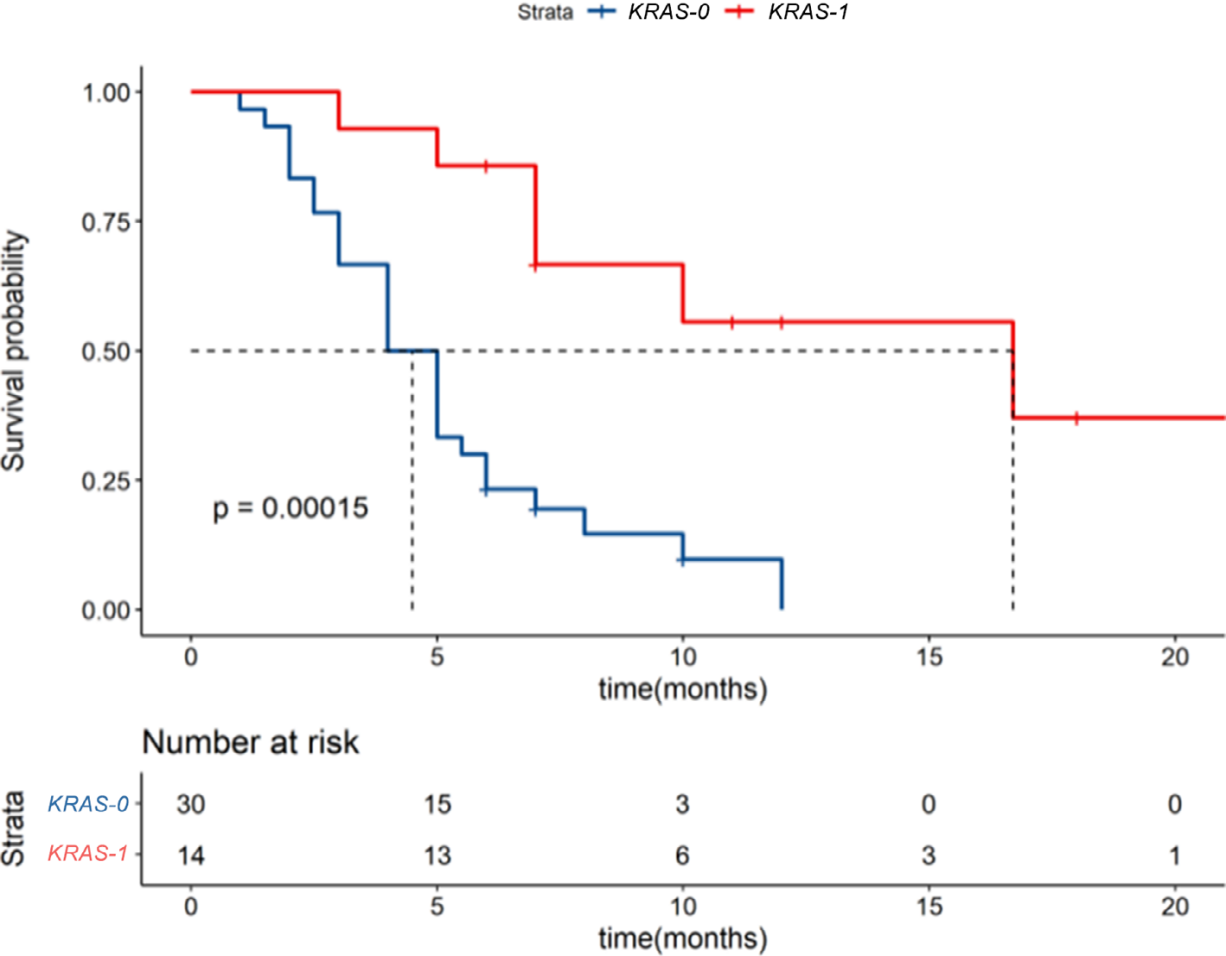
The Kaplan‐Meier method was used to draw a progression‐free survival curve for NSCLC patients with or without the *KRAS* gene mutation (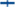) Strata, (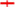) *KRAS* = 0, (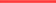 *KRAS* = 1).

Fig 6 shows the Kaplan‐Meier method for mapping the association between mutations in *KRAS* genes and progression‐free survival in NSCLC patients who received immunotherapy. It can be seen that the difference in progression‐free survival times between the mutant group and the nonmutated group is statistically significant (PFS) (*P* = 0.00015). The *KRAS* mutation is therefore a favorable predictor of the long‐term benefit of immunotherapy in NSCLC patients.

**Figure 7:**
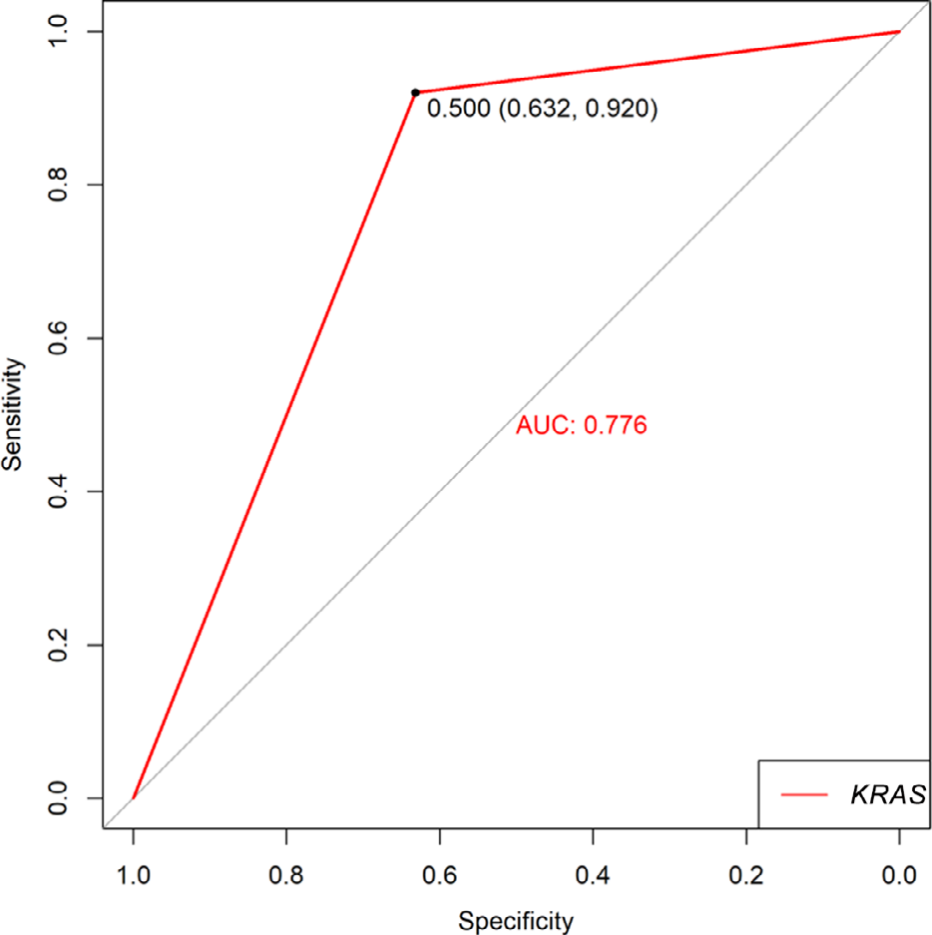
ROC curve of *KRAS* prediction outcome (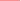) *KRAS*.

### KRAS mutation predicts the long‐term benefits of immunotherapy

The ROC curve was drawn based on whether the long‐term benefit (two‐category outcome) is the dependent variable and whether the *KRAS* mutation is used as a predictor, as shown in Fig 7.

The area under the curve is 0.776, and according to the principle of approximately equal exponential maximum, 0.5 is the most optimal cutoff value. At this time, the specificity is 0.632, and the sensitivity is 0.920. The probability of false negatives is 0.080, and the probability of false positives is 0.368, suggesting that the ability of the *KRAS* gene mutation to predict outcomes is moderately high.

## Discussion

Genetic changes in specific driver genes activate tumor cell proliferation to support tumor growth. It has been shown that certain oncogenic pathways also affect the immune system's recognition of tumors, especially T cell‐mediated recognition. Identifying lung adenocarcinoma subtypes with carcinogenic drivers has revolutionized the treatment of NSCLC. *KRAS* mutations in solid tumors appear in 90% of pancreatic cancer cases, 10%–15% of lung cancer (mainly NSCLC) cases, and 30%–40% of colorectal cancer cases.

The *KRAS* mutation is the second most important oncogene‐driven mutation in lung adenocarcinoma, with *KRAS* missense mutations^9^ found in codons 12 and 13 appearing in more than 95% of cases. Unlike *EGFR* mutations, there is no gender difference with *KRAS* mutations, and they are more common in white populations than Asians, and in most patients who previously smoked or now smoke.^10^, ^11^ The biological and phenotypic heterogeneity of patients with *KRAS* mutations prevents the emergence of more effective treatment strategies for patients with *KRAS* mutations.^12^

Given that the activation of specific oncogenic pathways can have a broad effect on gene expression, the genetic makeup of cancer cells may have a significant impact on the immune tumor microenvironment (TME) by driving specific immune‐related pathways. This can be achieved by inducing immune checkpoints, secreting specific cytokines, or producing chemokines that recruit specific cell types.^13^ Recent studies have shown that *KRAS*‐mutant NSCLC expresses higher levels of PD‐L1 protein^14^, ^15^, ^16^ compared to corresponding wild‐type tumors. Therefore, it can be speculated that the most common smoking‐related mutation in lung adenocarcinoma, *KRAS*, can be used as an effective predictor of anti‐PD‐1/PD‐L1 immunotherapy.

Cinausero *et al*.^17^retrospectively analyzed 88 patients with locally advanced or metastatic nonsquamous NSCLC who received ICIs and found that patients with *KRAS* mutations had longer overall survival (OS) and PFS than patients with *KRAS* wild‐type, which was statistically significant. In addition, the presence of nonsynonymous *KRAS* mutations is associated with DCB. Dong *et al*.^8^ found that *KRAS*‐mutated tumors showed a significantly increased mutation load. *KRAS* mutations changed a group of genes involved in cell cycle regulation, DNA replication, and damage repair.

Public clinical trials and prospective observations of immunotherapy analysis have further confirmed that there is significant clinical benefit for patients with *KRAS* mutations to receive PD‐1 inhibitor treatment. In the current study, 44 patients were tested for tumor gene mutations, and multiple statistical analysis methods (lasso regression + logistic regression + machine learning random forest algorithm) were used to find that patients with *KRAS* mutations benefited from PD‐1 blockade. However, Jeanson *et al*.^18^ found that 282 patients with advanced NSCLC who received immunotherapy exhibited no significant *KRAS* mutations or any other mutations that made a difference in the ORR, PFS, or OS rates.

The underlying mechanism by which patients with *KRAS*‐activated mutations may benefit from PD‐1 blockade remains unclear. Most studies suggest that *KRAS* mutations can enhance PD‐L1 expression, promote T cell infiltration, and enhance tumor immunogenicity. This is attributed to the association between smoking and the presence of *KRAS* mutations.^19^ Snjezana *et al*.^20^ analyzed 3026 patients and found that *KRAS* mutations occurred in 34% of smokers and 6% of never‐smokers, and the most common G > T conversion mutation in smokers was *KRAS* G12C. In addition, it was found that any history of smoking significantly increased the possibility of finding *KRAS* mutations in lung cancer, regardless of the number of years of smoking.

The permanent damage to DNA caused by tobacco carcinogens obtained during smoking is the main source of most *KRAS* mutated lung adenocarcinomas. Therefore, the likelihood of *KRAS* mutations in lung cancer patients is determined by the number of years of smoking and does not significantly decrease over time after quitting. Dong *et al*.^8^ found a significant increase in the mutation load in *KRAS‐*mutant tumors, and also observed that *KRAS* mutations can disrupt DNA repair, especially in mismatch repair (MMR), which supports the idea that MMR deficiency can be a favorable factor for PD‐1 blockade. Therefore, the *KRAS* gene mutation may be a potential predictor of the success of immunotherapy involving the blockage of PD‐1.

In conclusion, the mutation of the *KRAS* gene in tumor tissues is an independent predictor of the long‐term benefit of immunotherapy, with strong predictive ability.

## Disclosure

No authors report any conflict of interest.

## Supporting information


**Appendix** **S1:** Supplementary Information
