# Supplementary figures and images for: Relationship between the efficacy of immunotherapy and characteristics of specific tumor mutation genes in non‐small cell lung cancer patients
Source: Thorac Cancer. 2020 Apr 27;11(6):1647–54. doi: 10.1111/1759-7714.13447 (PMC7262886; doi:10.1111/1759-7714.13447)

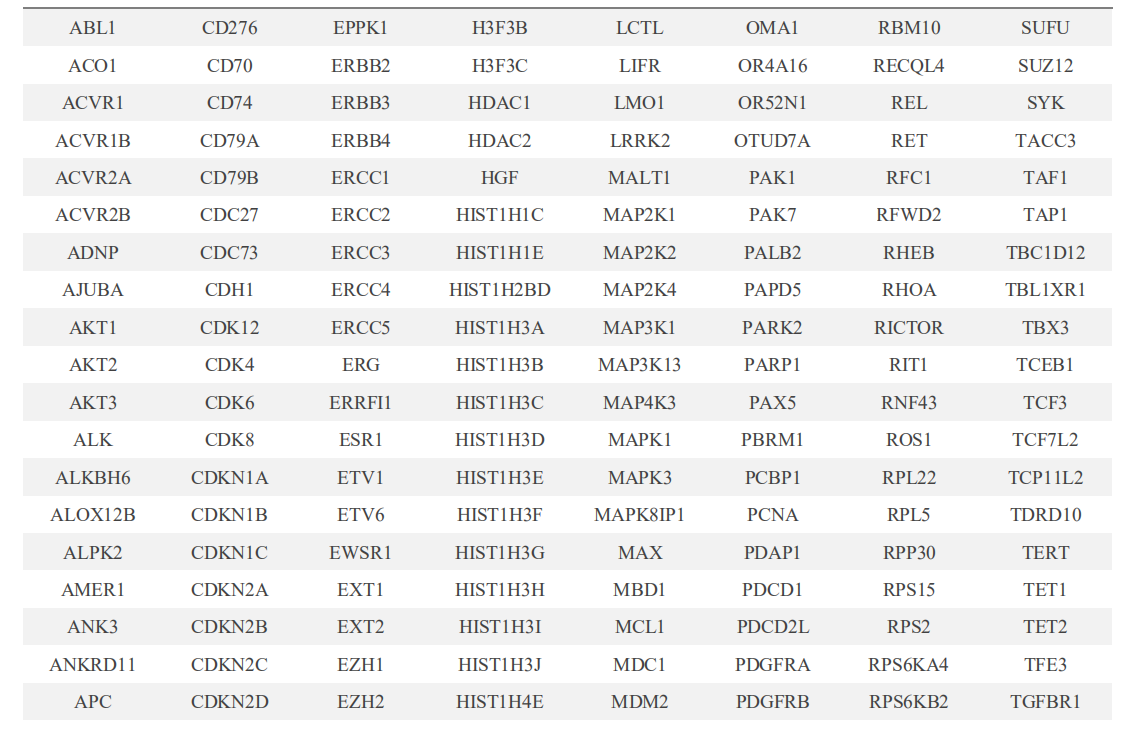


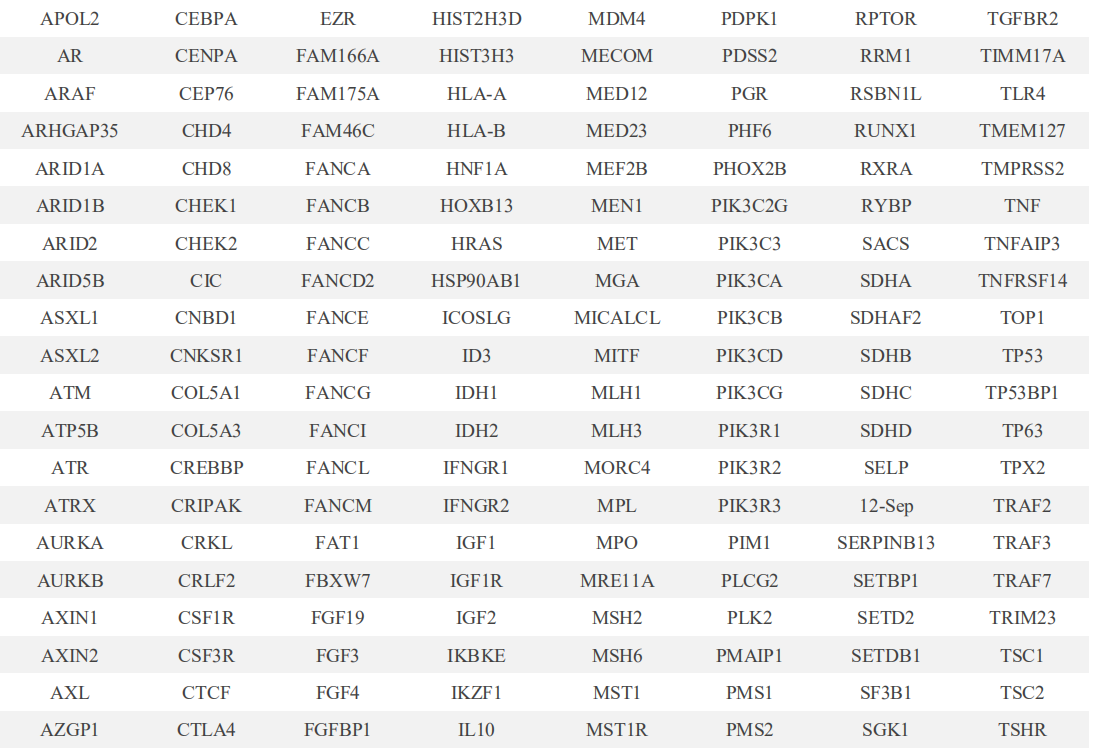


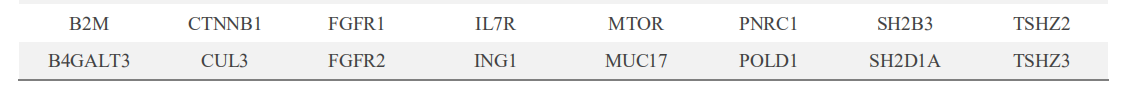


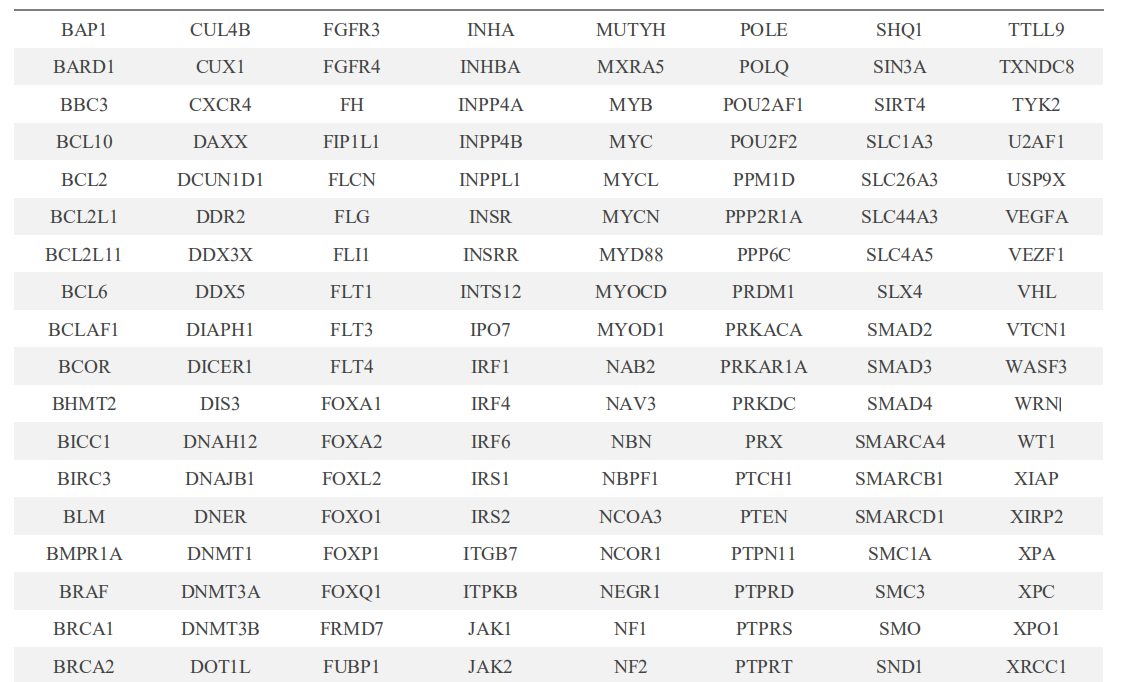


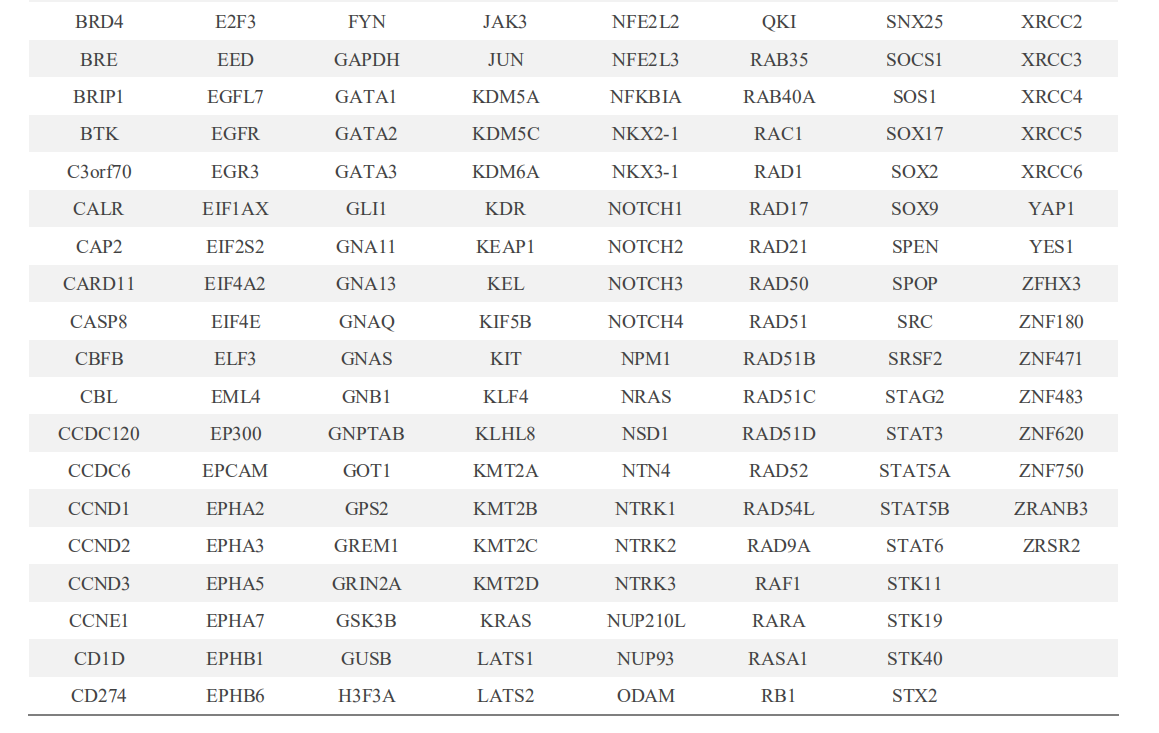

Supplement: Supplementary file 1 — Appendix S1: Supplementary Information [file TCA-11-1647-s001.docx]
